# History of Maternal Mortality in the City of Ribeirão Preto, in its Regional Health Department, and in the State of São Paulo after the Establishment of the Maternal Committees from 1998 to 2017

**DOI:** 10.1055/s-0040-1719143

**Published:** 2021-01-28

**Authors:** Aderson Tadeu Berezowski, Antonio Luiz Rodrigues Júnior

**Affiliations:** 1 Faculdade de Medicina de Ribeirão Preto, Universidade de São Paulo, Ribeirão Preto, SP, Brazil

**Keywords:** maternal mortality, health regionalization, mother and child healthcare, morte materna, regionalização da saúde, atenção materno-infantil

## Abstract

**Objective** To describe the evolution of maternal mortality right after the establishment of maternal death committees in the region of the city of Ribeirão Preto, state of São Paulo, Brazil.

**Methods** The present study describes the spatial and temporal distribution of maternal mortality frequencies and rates, using data from the state of São Paulo, the municipality of Ribeirão Preto, and its Regional Health Department (DRS-XIII) from 1998 to 2017. The present ecological study considered the maternal mortality and live birth frequencies made available by the Computer Science Department of the Brazilian Unified Health System (Departamento de Informática do Sistema Único de Saúde, DATASUS, in the Portuguese acronym)/Ministry of Health, which were grouped by year and political-administrative division (the state of São Paulo, the DRS-XIII, and the city of Ribeirão Preto). The maternal mortality rate (MMR) was calculated and presented through descriptive measures, graphs, and cartograms.

**Results** The overall MMR observed for the city of Ribeirão Preto was of 39.1; for the DRS-XIII, it was of of 40.4; and for the state of São Paulo, it was of 43.8 for every 100 thousand live birhts. During this period, the MMR for the city of Ribeirão Preto ranged from 0% to 80% of the total maternal mortalities, and from 40.7% to 47.2% of live births in the DRS-XIII. The city of Ribeirao Preto had an MMR of 76.5 in 1998and 1999, which decreased progressively to 12.1 until the years of 2012 and 2013, and increased to 54.3 for every 100 thousand live births over the past 4 years. The state of São Paulo State had an MMR of 54.0 in 1998–1999, which varied throughout the study period, with values of 48.0 in 2008–2009, and 54.1 for every 100 thousand live births in 2016–2017. Several times before 2015, the city of Ribeirão Preto and the DRS-XIII reached the Millennium Goals. Recently, however, the MMR increased, which can be explained by the improvement in the surveillance of maternal mortality.

**Conclusion** The present study describes a sharp decline in maternal death in the region of Ribeirão Preto by the end of 2012–2013, and a subsequent and distressing increase in recent years that needs to be fully faced.

## Resumo

**Objetivo** Descrever a evolução da mortalidade materna após a instituição dos comitês de morte materna na região de Ribeirão Preto.

**Métodos** Este estudo descreveu a distribuição espacial e temporal das frequências e da razão de mortalidade materna, utilizando dados do estado de São Paulo, do Departamento Regional de Saúde de Ribeirão Preto (DRS-XIII), e do município de Ribeirão Preto, no período de 1998 a 2017. O estudo ecológico considerou frequências de mortes maternas e de nascidos vivos disponibilizadas pelo Departamento de Informática do Sistema Único de Saúde (DATASUS)/Ministério da Saúde, que foram agrupadas por ano e pela referida divisão político-administrativa. A taxa de mortalidade materna (TMM) foi calculada e apresentada por medidas descritivas, gráficos e cartogramas.

**Resultados** O total observado para o município de Ribeirão Preto foi uma TMM de 39,1; para o DRS-XIII, TMM de 40,4; e, para o estado de São Paulo, uma TMM de 43,8 por 100 mil habitantes. No período do estudo, a RMM do município de Ribeirão Preto variou de 0% até 80,0% do total de mortes maternas, e de 40,7% a 47,2% dos nascidos vivos no DRS-XIII. O município de Ribeirão Preto apresentou TMM de 76,5 no biênio 1998–1999, que progressivamente diminuiu para 12,1 em 2012–2013, e aumentou para 54,3 por 100 mil habitantes nos últimos 4 anos. O estado de São Paulo apresentou TMM de 54,0 em 1998–1999, tendo variado ao longo do período com valores de 48,0 no período 2008–2009, e 54,1 no período 2016–2017. Várias vezes antes de 2015, o município de Ribeirão Preto e o DRS-XIII atingiram as Metas do milênio. Recentemente, porém, a TMM aumentou, o que pode ser explicado pela melhoria da vigilância da mortalidade materna.

**Conclusão** O estudo descreveu um acentuado declínio da morte materna na região de Ribeirão Preto até o final do biênio 2012–2013, e um subsequente e aflitivo aumento em anos recentes, que precisa ser enfrentado.

## Introduction

Maternal mortality is an important public health problem that must be addressed with effective and permanent measures, and its determinants are directly associated with the organization of the healthcare service and the increased value of women in society. 1 Maternal mortality has long been the subject of international discussions and of the Brazilian health reform in the mid-1980s, highlighting the efforts of Anibal Faúndes, a Chilean working in Brazil who helped create the Comprehensive Women's Health Care Program (Programa de Assistência Integral à Saúde da Mulher, PAISM, in the Portuguese acronym). The program broke the traditional view that women's care should be centered on reproductive issues, which contributed to the creation of the Maternal Mortality Committees. 2 The Maternal Mortality Prevention Program of the Department of Health of the State of São Paulo, which was created in 1987, was a precursor of the Maternal Mortality Committees, established in 1988 with the creation of the Botucatu, Campinas, Marília, Ribeirão Preto, and São Paulo committees. Initially, the committees were located in the medical schools of those regions, and, based on their initial experience, the program was later disseminated throughout Brazil. 3 4 5 A progressive structuring of committees throughout the country then occurred, institutionalizing the program at the national, state, regional, municipal and local levels. 6

In 1997, the report of maternal mortalities became compulsory, and groups were established to study the subject in depth, identify causality, understand avoidability, and guide the regional structuring of comprehensive healthcare for women, especially maternal healthcare, aimed at tackling the problem and finding solutions. 7 The objective was to support specialized groups to discuss on a case-by-case basis women's deaths due to pregnancy and the pregnancy-puerperal cycle, and provide managers with the information needed for decision-making toward a reduction in maternal mortality. In the Ribeirão Preto region, where one of the first committees was created and where the headquarters of the Regional Health Department (DRS-XIII) are located, maternal mortality is a serious public health issue, similar to the situation in the rest of the country.

The present study aimed to produce a spatial and temporal distribution of maternal mortality, describing the epidemiological phenomena by considering as ecologic unities the state of São Paulo, the DRS-XIII, and the city of Ribeirão Preto, and using official data from 1998 to 2017.

## Methods

The present is an ecological study on maternal mortality from 1998 and 2017, which used public and official data provided by the Computer Science Department of the Brazilain Unified Health System (Departamento de Informática do Sistema Único de Saúde, DATASUS, in the Portuguese acronym)/Ministry of Health. Maternal mortality and live birth frequencies were grouped by year (time) and by the political division of state of São Paulo's Administrative Office into Regional Departments of Health (spatial). Maternal mortality and live birth frequencies were collected and organized by year from 1998 to 2017; the ecological unities were defined as the state of São Paulo (645 municipalities), the DRS-XIII, and the city of Ribeirão Preto. The data were collected in September 2019. The maternal frequencies were rearranged by biennium because the yearly frequencies were too small, and data variation was great. The maternal mortality rates (MMRs) were obtained by taking the counts of deaths of pregnant women plus the counts of deaths of women that happened up to the 42nd day after delivery by causes related to pregnancy or not, with the exception of accidental deaths; theses sums were divided by the total live births according to temporal and spatial strata, and their results were expressed for every 100 thousand live births. 8 9

Tables, charts, and cartograms were used to produce spatial and temporal information using the R software (R Foundation for Statistical Computing, Vienna, Austria). Data from 2018 on were not included due to the delay in data verification and formalization by the DATASUS. The cartograms helped in the geographical interpretation of different municipalities in the DRS-XII, by considering the existence of any structure related to mother and child healthcare in each municipality of the DRS-XIII, according to the Brazilian National Registry of Healthcare Establishments (Cadastro Nacional de Estabelecimentos de Saúde, CNES, in the Portuguese acronym).

The present study used public secondary data and was conducted according to the ethics criteria establish by Resolution No. 510/16, Article 1, sole paragraph, items II, III, IV, and V of the Brazilian National Health Council.

## Results

The Table 1 presents the annual maternal mortality from 1998 to 2017 for the city of Ribeirão Preto, the DRS-XIII, and the state of São Paulo. Throughout the period, Ribeirão Preto represented 1.1% of the total maternal mortalities and 1.3% of the total live births in the state of São Paulo; the DRS-XIII represented 2.6% and 2.9% of that total respectively. Ribeirão Preto had an MMR of 39.1, the DRS-XIII, of 40.4, and the state of São Paulo State, of 43.8 for every 100 thousand live births. Among the data reported for the DRS-XIII, in the period, 43.1% of maternal mortalities and 44.5% of live births occurred in Ribeirão Preto; the percentage of maternal mortality for this municipality ranged from 0% to 80.0%, and the percentage of live births, from 40.7% to 47.2%.

**Table 1:** Temporal and spatial frequency distribution of maternal mortality and live births from 1998 to 2017 in the city of Ribeirão Preto, the Regional Department of Health (DRS-XIII), and the state of São Paulo

Year	Ribeirão Preto		DRS-XIII		State of São Paulo	
	Maternal mortality	Live births	Maternal mortality	Live births	Maternal mortality	Live births
1998	6	7,660	10	18,802	386	693,413
1999	6	8,033	12	19,089	374	714,428
2000	3	7,880	4	18,836	275	687,779
2001	5	7,407	16	17,312	257	632,483
2002	5	7,646	9	17,096	248	623,302
2003	0	7,462	6	17,494	208	610,555
2004	1	7,600	6	17,763	214	618,080
2005	4	7,691	7	17,926	219	618,880
2006	1	7,395	4	16,743	246	603,368
2007	3	7,297	7	15,965	252	595,408
2008	4	7,668	8	16,966	246	601,795
2009	2	7,870	8	17,241	339	598,473
2010	2	8,141	3	17,971	271	601,352
2011	4	8,353	5	18,225	249	610,222
2012	0	8,272	3	17,840	227	616,608
2013	2	8,210	3	17,925	240	610,896
2014	2	8,628	4	18,669	263	625,687
2015	3	8,834	12	18,939	311	634,026
2016	5	8,271	9	17,539	308	601,437
2017	4	8,322	8	17,858	348	611,803
Total	62	158,640	144	356,199	5,481	12,509,995

**Fig. 1:**
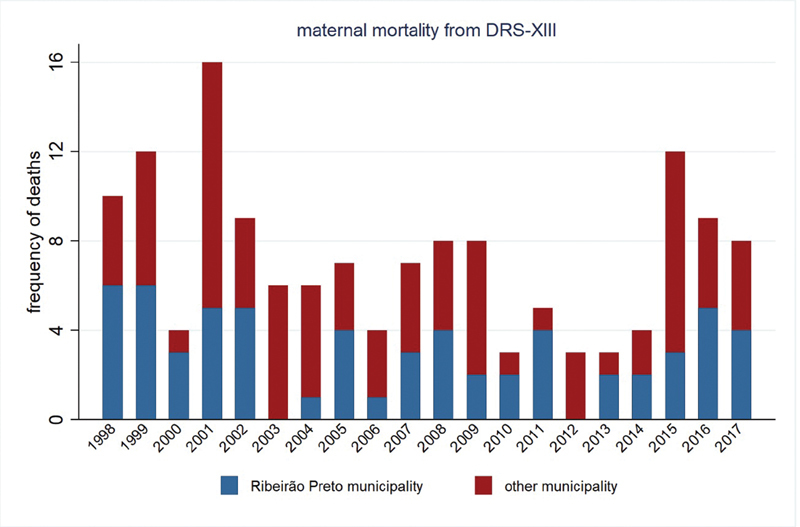
Stacked bar chart showing the yearly frequency of maternal mortalityies according to the place of residence: the city of Ribeirão Preto and the other municipalities of the Regional Department of Health (DRS-XIII).

The Fig. 1 shows the number of cases of maternal mortality recorded by year in Ribeirão Preto and the other municipalities of the DRS-XIII. As one can see in the figure, Ribeirão Preto registers an expressive fraction of maternal mortality when compared with the other 25 municipalities of the DRS-XIII, which influenced similarities between the epidemiological patterns of Ribeirão Preto and the DRS-XIII.

**Fig. 2:**
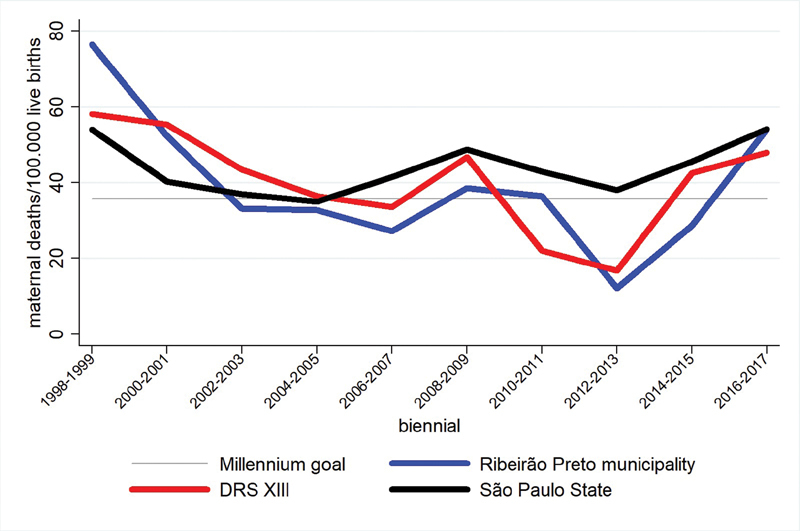
Biennial distribution of maternal mortalities rates (100 thousand live births) for Ribeirão Preto, the DRS-XIII, and the state of São Paulo.

The Fig. 2 shows biennial time series of MMRs which allowed a more clear observation than that of yearly rates, because of the aforementioned variation control. An expressive drop in MMRs was observed from 1998 on in Ribeirão Preto and in the DRS-XII. In 1998, Ribeirão Preto had an MMR of 76.5 for every 100 thousand live births, and this rate started to drop until 2012–2013, when the MMR was of 12.1, but unfortunately it begun to increase, reaching 54.2 over the past 4 years. The state of São Paulo did not show an expressive decrease in the maternal mortality: in 1998, the MMR was of 54.0; it increased and decreased until reaching 48.0 in 2008–2009; and it rose again to 54.1 in 2016–2017.

**Fig. 3:**
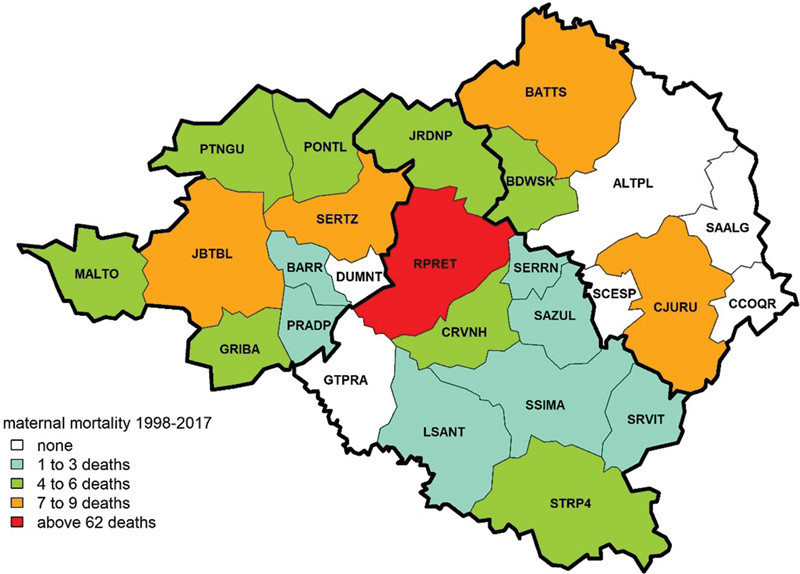
Spatial distribution of maternal mortality frequencies among the municipalities of the DRS-XIII from 1998 to 2017 by empirical classification legend.

The Fig. 3 shows the spatial distribution of frequencies of maternal mortality using empirical cut points to create captions, because the quantile classification led to very confusing divisions. The current division enables the observation of municipalities without maternal mortality in the period, and the outlier influence of maternal counts from Ribeirão Preto. This illustration shows those municipalities that did not report maternal mortalities in the period, like Altinópolis (ALTPL), Santo Antonio da Alegria (SAALG), Cássia dos Coqueiros (CCOQR), Dumont (DUMNT), Guatapará (GTPRA), and Santa Cruz da Esperança (SCESP); in addition, the municipalities who registered few maternal deaths were Barrinha (BARR), Pradópolis (PRADP), Luis Antonio (LSANT), São Simão (SSIMA), Santa Rosa de Viterbo (SRVIT), Cravinhos (CRVNH), Serra Azul (SAZUL), Serrana (SERRN), Brodowski (DBWSK), and Jardinópolis (JRDNP).

**Fig. 4:**
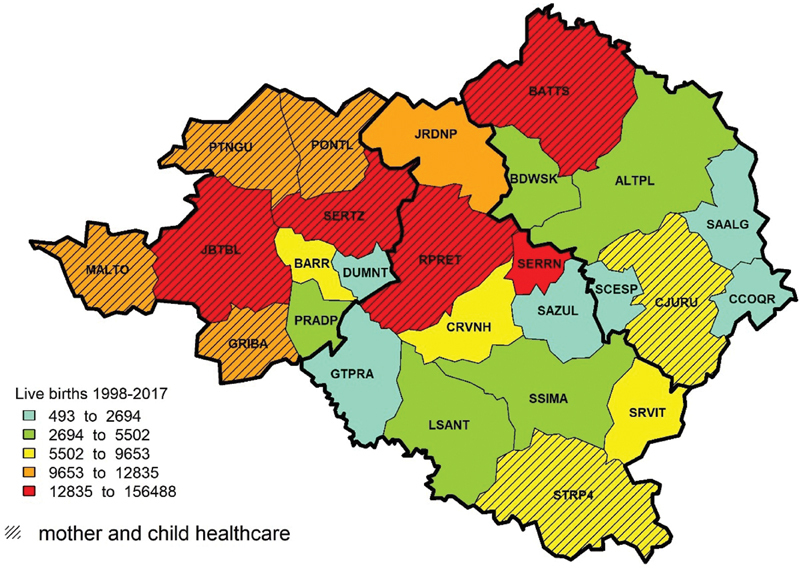
Spatial distribution of live births for each municipality of the DRS-XIII from 1998 to 2017, overlaid by places where there were mother and child healthcare services registered at the CNES.

The Fig. 4 shows the frequencies of live births in the 26 municipalities of the DRS-XIII that were divided by quintile class; in this case, the caption division did not produce misinterpretations on risk. By overlaying the existence of mother and children healthcare services registered by the CNES, the illustration highlights those places with expressive maternal deaths where there was no proper assistance for pregnant women. The illustration also shows that several municipalities are far from Ribeirão Preto, and sometimes are located near the border of the DRS-XIII. Among all municipalities of the DRS-XIII, sixteen did not offer specialized mother and child healthcare services. The figure also shows that the municipalities with the largest number of live births are located in the north of the DRS-XIII, and the municipalities located in the south and east of the DRS-XIII have lower live birth rates; among these, only Cajuru (CJURU) and Santa Rita do Passa Quatro (STRP4) offered specialized mother and child healthcares. Both figures are complementary, as the indicator is calculated by the number of maternal mortalities and the total live births. The cartogram of maternal mortality shows the municipalities where pregnant women get exposed to the determining environmental factors, where they develop their lifestyles, and where they access mother and child healthcare services, when they exist.

## Discussion

The United Nations (UN) sponsored the Millennium Summit in 2000, which was attended by 191 correspondents of the member states, and the signatory countries agreed to eight goals to be achieved until 2015, aiming the 21st century human development; they became known as Millennium Development Goals (MDGs). 10 The fifth MDG involves the reduction of maternal mortality by 75.0%, based on 1990 indicators. 11 Brazil presented 143 maternal mortalities per 100 thousand live births in 1990; by considering the known underreporting at that time and the need for adjusted estimates, the MDG stated that the achievement of an MMR of 35.8 in 2015 would demand an annual hypothetical reduction of 4.3 unities per year. 12 In 2015, the MMR in Brazil was of 62.0. 13 By using the data of the present study, the state of São Paulo State had an MMR of 49.1, which is in line with the study by Morse et al; 11 the DRS-XIII had an MMR of 63.4; and Ribeirão Preto, an MMR of 34.0. According to the data in Table 1 , Ribeirão Preto reached the MDG for the MMR in 2004 (13.2), in 2006 (13.5), in 2009 (25.4), in 2010 (24.6), in 2012 (0.0), in 2013 (24.4), in 2014 (23.2), and in 2015 (34.0). The DRS-XIII reached the MDG for the MMR in 2000 (21.2), in 2003 (34.3), in 2004 (33.8), in 2006 (23.9), in 2010 (16.7), in 2011 (27.4), in 2012 (16.8), in 2013 (16.7), and in 2014 (21.4). Finally, the state of São Paulo reached the MDG for the MMR in 2003 (34.1), in 2004 (34.6), and in 2005 (35.4).

Thinking about the range of frequencies throughout the study period ( Fig. 1 ), the prevention for maternal mortality would not get the effectiveness and it would argue politics out about pregnancy protection programs and actions. The estimates should be taken by facing the precision of binary variables (poisson, binomial or logistic) for rare events into small populations (overdispersion) while the epidemiological analysis is to be performed. If maternal deaths in Ribeirão Preto were observed as absolute counts (poisson random variable), in the years 2003 and 2012, this ecologic unity showed none, while in the corresponding years right before they were high, and this instability arose the burden of random effect along the period of study; if such variable was to be observed as proportion in regard total counts into DRS-XIII (binomial or logistic random variable), the overdispersion would also be presented as well as source of confounding because statistical estimation property.

After 1998, the MMR in Ribeirão Preto decreased until its lowest estimate in 2012–2013 (12.1), and then it increased again. By coincidence, the decrease in the MMR occurred before the implementation of the Regional Maternal Mortality Committee, so there is no association between them. As a matter of fact, the MMR had been decreasing over the previous decades through the control of the environmental conditions on broad public health programs and actions, specially in primary care, despite the fact that the implementation of the Maternal Mortality Committee meant an important strategy to boost that decrease. The Fig. 2 also shows a conservative trend in the epidemiologic curve for the state of São Paulo for the past 20 years. There was no significant decrease, but a stable fluctuation, as the MMRs at the beginning and at the end of the period were similar.

Nevertheless, recent increases in MMRs were observed, and they can be explained by two conjectures : 1) the organization of mother and child healthcare is a very meaningful environmental determinant, and 2) the improvement in the technical standardization of protocol detection arose the likelihood of maternal mortality reality. These conjectures would be applicable alone or combined.

The Fig. 3 shows 6 municipalities with no maternal mortality reports, a low rate of live births, and no mother and child healthcare services. The absence of specialized healthcare may be explained by economic reasons, as the costs of maintaining it are high, while the demand for it is low. The Fig. 4 shows that the municipalities located to the south and east of the DRS-XIII had the lowest absolute birth rates and did not have mother and child specialized care, except for two municipalities (Cajuru and Santa Rita do Passa Quatro). Thus, these municipalities are more vulnerable to maternal mortality and, because of their small populations, the difficulties to establish and maintain specialized mother and child healthcare services are great. The alternative for those municipalities, therefore, is the improvement of primary healthcare by focusing on assistance, communication, and transportation systems, as a result of intermunicipality planning and acts for sharing healthcare resources.

By the times of economic crisis, it must concern the costs to maintain maternity ward because scarce resources in municipalities, and Regional Intermanager Commission (RIC: “Comissão Intergestora Regional” in Portuguese), public or public-private partnerships, or even by management contracts with private institutions on a complementary basis, may contribute to the mother and child healthcare. If the establishment and maintenance of mother and child healthcare institutions is unfeasible due to economic reasons, emergency transportation and communication systems by telemedicine should be prioritized, so that women at high risk will be rapidly identified, and the regional assistance can react promptly. The professional training must be prevention-based and the Maternal Mortality Committee should contribute to the treatment protocols and specific clinical guidelines for the risk of maternal mortality.

Maternal mortality surveillance is the set of actions that enables the identification, detection, and prevention of the outcome. However, the Maternal Mortality Committee responsibility is not mandatory, but assessorial, including the case investigation, the establishment of critical learning, the determination of avoidability, the assessment of quality indicators, the strategy for the identification and the implementation, as well as the contribution to the sensitization of managers. 14

The Maternal Mortality Committees are supported by government institutions and the civil society, and have educational, non-coercive, and non-punitive attributions, keeping the confidentiality of issues discussed internally. In addition, while its membership is legally constituted, the RIC representatives should be concerned, as they play executive contracts and they may contribute for these issues involving mother and child healthcare.

## Conclusion

The present study provided a historical overview of maternal mortality in the state of São Paulo, the DRS-XIII, and the city of Ribeirão Preto after the implementation of the Maternal Mortality Committees in 1998. The positive impact on the fight against maternal mortality on the part of Ribeirão Preto and the DRS-XIII can be clearly observed, since, throughout the study period, they presented low MMRs and achieved the MDGs several times. However, in recent years, an increase in MMRs has been observed, which would be explained by an improvement in case identification. As an important environmental determinant, mother and child healthcare services play a crucial role in the control of maternal mortality.
